# Adaptive Neural Backstepping Sliding Mode Heading Control for Underactuated Ships with Drift Angle and Ship-Bank Interaction

**DOI:** 10.1155/2020/8854055

**Published:** 2020-09-27

**Authors:** Xue Han

**Affiliations:** ^1^School of Navigation, Jimei University, Xiamen 361021, Fujian, China; ^2^National-Local Joint Engineering Research Center for Marine Navigation Aids Services, Jimei University, Xiamen 361021, Fujian, China; ^3^Fujian Shipping Research Institute, Xiamen 361021, Fujian, China; ^4^Xiamen Southeast International Shipping Research Center, Xiamen 361021, Fujian, China

## Abstract

In order to track the desired path under unknown parameters and environmental disturbances, an adaptive backstepping sliding mode control algorithm with a neural estimator is proposed for underactuated ships considering both ship-bank interaction effect and shift angle. Using the features of radial basis function neural network, which can approximate arbitrary function, the unknown parameters of the ship model and environmental disturbances are estimated. The trajectory tracking errors include stabilizing sway and surge velocities errors. Based on the Lyapunov stability theory, the tracking error will converge to zero and the system is asymptotically stable. The controlled trajectory is contractive and asymptotically tends to the desired position and attitude. The results show that compared with the basic sliding mode control algorithm, the overshoot of the adaptive backstepping sliding mode control with neural estimator is smaller and the regulation time of the system is shorter. The ship can adjust itself and quickly reach its desired position under disturbances. This shows that the designed RBF neural network observer can track both the mild level 3 sea state and the bad level 5 sea state, although the wave disturbance has relatively fast time-varying disturbance. The algorithm has good tracking performance and can realize the accurate estimation of wave disturbance, especially in bad sea conditions.

## 1. Introduction

In ship heading control, the steering motion makes the bow produce a small lateral drift angle and changes the fluid distribution on both sides of the hull. This will result in the difference between the actual motion direction and the expected ship heading. The current steering control algorithm ignores the effect of the drift angle, but the existence of the drift angle will increase overshoot and reduce the steering control accuracy and performance. In bad sea conditions, the model dynamic uncertainty, time-varying parameters, and drift angle correction need to be considered. The shift angle was proposed by Yu in 2008 [1]. Liu proposed a horizontal-plane cascade-steering model with drift angle and developed an adaptive backstepping control algorithm based on the dynamic surface control and Nussbaum gain techniques [2]. Lu designed a backstepping heading controller with a sideslip angle amendment and a robust adaptive control method combining the nonlinear disturbance observer to estimate the wave disturbance effectively in the yaw direction [3]. Sun proposed a guidance control scheme based on finite-time observers for path following control of underactuated unmanned vehicles subjected to time-varying large sideslip angle and unknown external disturbances [4].

For large ships, the channel width is narrow and the water depth is shallow. The maneuverability of ships navigating in restricted waters is quite different from that in open waters, mainly due to the influence of the bottom and bank wall, and the hydrodynamic force is usually greater. For the ships running near the shore, the effect of the quay wall is a potentially unsafe factor, which often leads to collision accidents caused by ships too close to the shore wall, resulting in casualties and a large number of property losses. Therefore, it is necessary to study the ship-bank interaction effect [5].

Recently, backstepping control is used for uncertain nonlinear systems to improve the global ultimate asymptotic stability. Chu proposed an adaptive global sliding mode fuzzy control using a radial basis function neural network based on the backstepping technique [6]. A RBF neural approximator was employed to estimate uncertainty. Wang deduced the control algorithm for the unmanned surface vehicle by the backstepping method with Lyapunov theory [7]. Liu proposed a backstepping adaptive dynamic sliding mode control method for the track tracking control system of underactuated surface ships [8]. Li proposed an adaptive backstepping sliding mode control method for a class of uncertain nonlinear systems with input constraints [9]. The explosion of complexity is avoided in the traditional backstepping design method by introducing a first-order filter. Chen applied an adaptive sliding mode controller based on the backstepping method to the robust trajectory tracking of the wheeled mobile manipulator [10]. The control algorithm adopted the backstepping method to improve the global ultimate asymptotic stability. Wang extended the iterative sliding mode controller based on reinforcement learning to bidirectional control, considering the trajectory tracking problem of Underactuated Ships under known and unknown conditions [11]. Disturbance, dynamic surface control technology, adaptive neural network, sliding mode control algorithm, and backstepping design method are combined to design neural network adaptive sliding mode control law bound for environmental disturbance. Meng proposed a robust sliding mode method for trajectory tracking of underactuated ships with uncertain parameters and time-varying disturbances [12]. In the dynamic loop, the trajectory tracking error is transformed into the steady surge and surge velocity error by using the backstepping technique. Wang used a RBF neural network to approximate the ship system function and external disturbance [13]. Lyapunov stability theory and backstepping method were used to design the controller of ship motion. Wen utilized the property of neural network compensation technique and backstepping method, to construct an adaptive output feedback controller [14]. Sun constructed an adaptive decentralized tracking controller via the backstepping method and neural network technique, where a sliding mode differentiator was presented to estimate the derivative of the virtual control law and reduce the complexity of the control scheme [15].

For dealing with systems with uncertainty, several methods have been used, such as fuzzy logic, neural network, and so on. Cheng presented a novel indirect neural observer with the ADALINE network incorporated into the conventional sliding mode term and utilized a radial basis function neural network approximation utilized to handle the system uncertainties [16]. Gajate presented the design and application of a novel transductive neurofuzzy inference method to control force in a high-performance drilling process [17]. Martín considered a neurofuzzy system, where fuzzy rules were obtained from input/output data [18]. The design of the control system was based on the internal model control paradigm. Kelly presented a fuzzy adaptation scheme for PD control with gravity compensation of robot manipulators [19]. It compensated for static friction in the robot joints and considered the real torque actuators capabilities to avoid torque saturation. Ramírez used a knowledge-based fuzzy controller, to keep the temperature as close to the set profile as possible. Such a controller was endowed with a set of 60 rule bases, which were dynamically switched depending on technological constraints and/or operating regions [20]. Guerra presented a procedure for digital twin-based optimization, in which the maximum absolute position error was minimized while maintaining accuracy with no significant increase in the control effort [21].

Sliding mode control is a branch of variable structure control proposed by Soviet scholars in the 1950s. Guo proposed a hybrid control algorithm based on backstepping control and nonsingular fast terminal sliding mode control to solve the tracking problem of the mobile robot [22]. Elmokadem proposed a robust control scheme for dynamic positioning and path tracking of the underactuated autonomous underwater vehicle (AUV) based on sliding mode control [23]. Sun proposed a new nonlinear robust adaptive control scheme with sliding mode control to track the desired path generated by the logic virtual ship under unknown parameters and environmental disturbances [24]. In order to solve the problem that the ship speed state vector cannot be measured, Wang designed an observer based on an output feedback sliding mode controller [25]. An adaptive sliding mode controller based on RBF neural network is designed to solve the problem of underactuated ship trajectory tracking with uncertain model parameters and large external disturbance. To solve the problem of track tracking of a three degree of freedom underactuated ship, Dai proposed an adaptive iterative sliding mode control method based on Reinforcement Learning [26]. Sui proposes a neural network sliding mode control strategy based on a dynamic virtual ship algorithm, which is controlled under the condition of model uncertainty and external disturbances such as wind, wave, and current [27]. To solve the problem of automatic berthing of 3-DOF ships, Zou proposed an iterative sliding mode control based on chaos particle swarm optimization [28].

However, how to design a robust controller for ships is still a challenging problem because of model errors, parameter changes, and external disturbances. First of all, the existence of a drift angle is usually ignored in course control, but the actual drift angle is not zero, which will cause a drift angle difference between the actual motion direction of the ship and the expected course. If not corrected, the performance of course control will be reduced. Secondly, the effect of ship-bank interaction is a common phenomenon in berthing, crossing bridge, and near-shore navigation. The large ship-bank interaction effect can even lead to ship capsizing. However, in ship motion control, the existence of the ship-bank interaction effect is usually ignored. Thirdly, the sudden disturbance will cause the control force to be too large, which will lead to the excessive driving power of the actuator. Any actuator has a certain range of execution; once the input exceeds the limit value, it will affect the operation of the actuator, resulting in system performance degradation, affecting the control effect, and maintaining too large control input for a long time will also increase the loss of rudder.

In this paper, the small angle approximation and slow time-varying assumption are avoided. The unknown disturbances such as drift angle and ship-bank interaction effect are dynamically estimated by the neural network. The time-varying large drift angle is accurately compensated by using the weight parameter adaptive law of the RBF neural network. Based on backstepping control and adaptive sliding mode technology, the surge speed and heading tracking controllers are designed, respectively, to realize the precise path tracking control with unknown time-varying large drift angles and the influence of the bank wall effect. The stability of the closed-loop system is proved by the Lyapunov theory. The experimental results show that the algorithm can reduce the heading error, the overshoot, adjustment time, and the control torque of the actuator. It can improve the robustness and economy of path tracking control for underactuated ships under unknown sea conditions.

## 2. Mathematical Model of Underactuated Ship

### 2.1. Mathematical Model of Underactuated Ship

**Figure 1:**
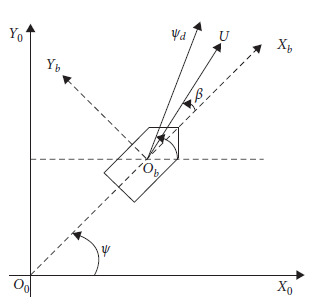
Ship motion.

The coordinate system is established as follows: The stationary observer on the shore is defined as the origin *O*_0_. The positive east direction is defined as the *X*_0_ axis and the positive north direction is defined as the *Y*_0_ axis. The three-degree-of-freedom motion of surge, sway, and yaw are considered. The motion model of the dynamic positioning system is shown in Figure 1. *η* denotes the position and attitude vector of the ship. *v* denotes the velocity vector of the ship. *u* denotes the surge velocity. *v* denotes the sway velocity. *r* denotes the yaw velocity. *x* denotes the position in surge direction. *y* denotes the position in sway direction. *ψ* denotes the heading angle. *ψ*_*d*_ denotes the desired heading angle. *β* denotes the shift angle.

The mathematical model of the dynamic positioning ship is as follows:

(1) $$\begin{array}{c} \dot{\eta }=R\left(\psi \right)\upsilon ,\\ \eta ={\left[\begin{array}{ccc} x & y & \psi \end{array}\right]}^{T},\\ \upsilon ={\left[\begin{array}{ccc} u & v & r \end{array}\right]}^{T}, \end{array}$$

where *R* is the rotation matrix, which can be calculated as follows:

(2) $$\begin{array}{c} R=\left[\begin{array}{ccc} \cos\psi & -\sin\psi & 0\\ \sin\psi & \cos\psi & 0\\ 1 & 1 & 1 \end{array}\right], \end{array}$$

The dynamic models of underactuated ships can be calculated as follows:

(3) $$\begin{array}{c} \left\{\begin{array}{c} \dot{x}=u\cos\psi -v\sin\psi ,\\ \dot{y}=u\sin\psi +v\cos\psi ,\\ \dot{\psi }=r. \end{array}\right. \end{array}$$

The velocity equation is as follows:

(4) $$\begin{array}{c} \left\{\begin{array}{c} \dot{u}=\frac{m_{22}}{m_{11}}vr-\frac{d_{11}}{m_{11}}u+\frac{1}{m_{11}}\left(\tau_{u}+F_{wx}+F_{cx}+F_{sx}+F_{xb}\right),\\ \dot{v}=-\frac{m_{11}}{m_{22}}ur-\frac{d_{22}}{m_{22}}v+\frac{1}{m_{122}}\left(F_{wy}+F_{cy}+F_{sy}\right),\\ \dot{r}=\frac{m_{11}-m_{22}}{m_{33}}uv-\frac{d_{33}}{m_{33}}r+\frac{1}{m_{33}}\left(\tau_{r}+N_{wc}+N_{c}+N_{s}+N_{b}\right), \end{array}\right. \end{array}$$

where *F*_*wx*_ is the force of wind disturbance along the surge direction. *F*_*wy*_ is the force of wind disturbance along the sway direction. *N*_*wc*_ is the torque of wind disturbance along the yaw direction. *F*_*cx*_ is the force of current disturbance along the surge direction. *F*_*cy*_ is the force of current disturbance along the sway direction. *N*_*c*_ is the torque of current disturbance along the yaw direction. *F*_*sx*_ is the force of wave disturbance along the surge direction. *F*_*sy*_ is the force of wave disturbance along the sway direction. *N*_*s*_ is the torque of wave disturbance along the yaw direction. *F*_*xb*_ is the force of ship-bank interaction effect along the surge direction. *N*_*b*_ is the torque of ship-bank interaction effect along the yaw direction. *τ*_*u*_ denotes the control input along the surge direction. *τ*_*r*_ denotes the control input along the yaw direction. *m*_11_, *m*_22_, *m*_23_, *m*_32_, *m*_33_ are elements of the matrix composed of weight inertia and hydrodynamic additional inertia. *d*_11_, *d*_22_, *d*_23_, *d*_32_, *d*_33_ are elements of linear hydrodynamic damping parameter matrix.

Denote the following:

(5) $$\begin{array}{c} f_{u}=\frac{1}{m_{11}}\left(F_{wx}+F_{cx}+F_{sx}+F_{xb}\right),\\ f_{v}=\frac{1}{m_{122}}\left(F_{wy}+F_{cy}+F_{sy}\right),\\ f_{r}=\frac{1}{m_{33}}\left(N_{wc}+N_{c}+N_{s}+N_{b}\right). \end{array}$$

Then (4) can be written as follows:

(6) $$\begin{array}{c} \left\{\begin{array}{c} \dot{u}=\frac{m_{22}}{m_{11}}vr-\frac{d_{11}}{m_{11}}u+\frac{1}{m_{11}}\tau_{u}+f_{u},\\ \dot{v}=-\frac{m_{11}}{m_{22}}ur-\frac{d_{22}}{m_{22}}v+f_{v},\\ \dot{r}=\frac{m_{11}-m_{22}}{m_{33}}uv-\frac{d_{33}}{m_{33}}r+\frac{1}{m_{33}}\tau_{r}+f_{r}. \end{array}\right. \end{array}$$

In the system model, the number of system state variables is 3 and the number of input control variables is 2, so the system is underactuated.

### 2.2. Mathematical Model of Environmental Disturbing Force

The disturbance of external environment includes the disturbance of wind, sea wave, and current.

#### 2.2.1. Wind Disturbance Model

According to Isherwoodʼs research, the force and torque of wind disturbance can be calculated as follows:

(7) $$\begin{array}{c} \left\{\begin{array}{c} F_{wx}=\frac{1}{2}C_{X}\left(\gamma_{r}\right)\rho_{a}A_{f}V_{r}^{2},\\ F_{wy}=\frac{1}{2}C_{Y}\left(\gamma_{r}\right)\rho_{a}A_{s}V_{r}^{2},\\ N_{wc}=\frac{1}{2}C_{N}\left(\gamma_{r}\right)\rho_{a}A_{s}L_{oa}V_{r}^{2}, \end{array}\right. \end{array}$$

where *C*_*X*_, *C*_*Y*_ are the wind coefficient. *C*_*N*_ denotes the wind moment coefficient. *A*_*f*_ denotes the projection area above the water line. *A*_*s*_ denotes the projection area on the side. *L*_*oa*_ denotes the total length of the ship. *ρ*_*a*_ denotes the air density.

#### 2.2.2. Current Disturbance Model

The forces and moments acting on ships by ocean currents can be calculated as follows:

(8) $$\begin{array}{c} \left\{\begin{array}{c} F_{cx}=\frac{1}{2}\rho A_{fw}V_{C}^{2}C_{x}\left(\beta \right),\\ F_{cy}=\frac{1}{2}\rho A_{sw}V_{C}^{2}C_{y}\left(\beta \right),\\ N_{c}=\frac{1}{2}\rho A_{fw}LV_{C}^{2}C_{n}\left(\beta \right), \end{array}\right. \end{array}$$

where *F*_*cx*_, *F*_*cy*_ denote the longitudinal force and the transverse force produced by the current. *N*_*c*_ is the moment produced by the current. *V*_*c*_ represents the velocity of the current. *A*_*fw*_ denotes the orthographic projection area of the ship underwater. *A*_*sw*_ denotes the side projection area of the ship underwater. *L* denotes the length of the ship's waterline. *β* denotes the drift angle. *ρ* denotes the density of the sea water. *C*_*x*_, *C*_*y*_, *C*_*n*_ denote the longitudinal flow force coefficient, transverse flow force coefficient and moment coefficient, respectively.

#### 2.2.3. Wave Disturbance Model

The forces and moments produced by wave disturbance can be calculated as follows:

(9) $$\begin{array}{c} \left\{\begin{array}{c} F_{sx}=\frac{1}{2}\rho La^{2}\cos\chi C_{xw}\left(\lambda \right),\\ F_{sy}=\frac{1}{2}\rho La^{2}\sin\chi C_{yw}\left(\lambda \right),\\ N_{s}=\frac{1}{2}\rho L^{2}a^{2}\sin\chi C_{nw}\left(\lambda \right), \end{array}\right. \end{array}$$

where *a* denotes the average wave amplitude. *χ* denotes the encounter angle. *C*_*xw*_, *C*_*yw*_, *C*_*nw*_ denote the longitudinal wave drift force, transverse wave drift force, and moment coefficient, respectively. *λ* is the length of the wave.

#### 2.2.4. Ship-Bank Interaction Effect

**Figure 2:**
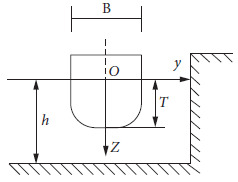
Ship-bank interaction effect.

When the ship is sailing near the bank of a channel or the pier of a bridge, the water flow near the bank accelerates and the pressure decreases. This results in the additional force which makes the ship close to the bank. This force is called the bank suction. Shore suction may cause the ship to touch the shore. At the same time, there is a moment which makes the bow deviate from the shore; that is, the shore thrust moment. Bank suction and bank thrust moment are generally called ship-bank interaction effects. The ship-bank interaction effect of a vertical wall is shown in Figure 2.

The force and moment of a vertical bank are calculated by Norrbin's formula [29].

(10) $$\begin{array}{c} F_{xb}=\rho C_{b}Bdu^{2}\eta_{0}\left[0.0925+0.327{\left(\frac{T}{h}\right)}^{2}\right],\\ N_{b}=-\rho C_{b}LBdu^{2}\eta_{0}\left[0.0025+0.0755{\left(\frac{T}{h}\right)}^{2}\right], \end{array}$$

where *ρ* is the water density. *C*_*b*_ is the block coefficient. *T* is the draught. *h* is the depth of the water. *L* is the length of the ship. *B* is the width of the ship. *η*_0_ is the ratio of ship width to distance between ship and shore.

## 3. Neural Backstepping Sliding Mode Control

### 3.1. RBF Neural Network

In 1985, Powell proposed a radial basis function (RBF) method for multivariate interpolation. The most frequently used Radial Basis Function is the Gauss function:

(11) $$\begin{array}{c} \phi_{i}\left(x\right)=e^{-\left({\left\|x-\mu_{i}\right\|}^{2}|/|2\sigma^{2}\right)},\\ \phi =\left[\phi_{1},\phi_{2},\ldots ,\phi_{P}\right], \end{array}$$

where *x* is the input vector. ‖*x*‖ denotes the Euclidean Norm of *x*. *φ*_*i*_ denotes the Radial Basis Function. *x*_*i*_ denotes the central vector of the function. *σ*_*j*_ denotes the width of the Radial Basis Function. *μ*_*i*_ denotes the threshold vector. *P* denotes the number of hidden layer nodes. *N* Denotes the number of input training samples. *y* denotes the output of the neural network:

(12) $$\begin{array}{c} y=W\left(n\right)\phi \left(x\left(n\right)\right),\\ W\left(n\right)=\left[w_{1}\left(n\right),w_{2}\left(n\right),\ldots ,w_{P}\left(n\right)\right]. \end{array}$$

**Figure 3:**
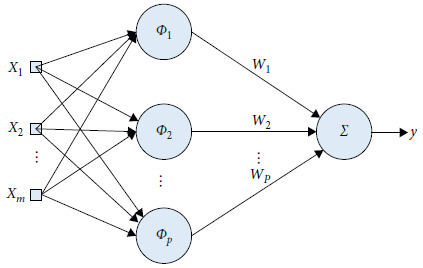
RBF structure.

Figure 3 shows the RBF structure.

In this system, the input of the RBF neural network is system state *x* and the output is ${\hat{f}}_{u}$ and ${\hat{f}}_{r}$. ${\hat{f}}_{u}$ is the approximation of *f*_*u*_ by RBF neural network. ${\hat{f}}_{r}$ is the approximation of *f*_*r*_ by RBF neural network.

### 3.2. Neural Backstepping Sliding Mode Control

The shift angle is computed as follows:

(13) $$\begin{array}{c} \beta =\arctan\frac{v}{u}. \end{array}$$

The heading error is computed as follows:

(14) $$\begin{array}{c} \psi_{e}=\psi -\psi_{d}+\beta . \end{array}$$

Taking the derivative of (14) can obtain the following:

(15) $$\begin{array}{c} {\dot{\psi }}_{e}=\dot{\psi }-{\dot{\psi }}_{d}+\dot{\beta }. \end{array}$$

Construct new variables:

(16) $$\begin{array}{c} z_{1}=\psi_{e}\\ =\psi -\psi_{d}+\beta , \end{array}$$

(17) $$\begin{array}{c} z_{2}=\dot{\psi }+k_{1}z_{1}-{\dot{\psi }}_{d}+\dot{\beta }\\ =r+k_{1}z_{1}-{\dot{\psi }}_{d}+\dot{\beta }, \end{array}$$

where *k*_1_ is a positive real number.

The following sliding surface functions are constructed:

(18) $$\begin{array}{c} s\left(t\right)=k_{2}z_{1}+z_{2}, \end{array}$$

where *k*_2_ is a positive real number.

The control rate is designed as follows:

(19) $$\begin{array}{c} \tau_{r}=d_{33}r-\left(m_{11}-m_{22}\right)uv+m_{33}\left[-{\hat{f}}_{r}-k_{1}{\dot{z}}_{1}+{\ddot{\psi }}_{d}-\ddot{\beta }-z_{1}+k_{2}\left(k_{1}z_{1}-z_{2}\right)-k_{3}s-\eta \mathrm{sgn}\left(s\right)\right], \end{array}$$

where *k*_2_ is a positive real number.

Denote ${\hat{f}}_{r}$ as the approximation of *f*_*r*_ by RBF neural network:

(20) $$\begin{array}{c} {\hat{f}}_{r}={\hat{W}}_{r}^{T}\phi , \end{array}$$

(21) $$\begin{array}{c} f_{r}=W_{r}^{T}\phi +\varepsilon , \end{array}$$

where *ε* is the approximation error of the RBF neural network.

Subtracting (20) from (21) can obtain the following:

(22) $$\begin{array}{c} f_{r}-{\hat{f}}_{r}=W_{r}^{T}\phi +\varepsilon -{\hat{W}}_{r}^{T}\phi \\ =\left(W_{r}^{T}-{\hat{W}}_{r}^{T}\right)\phi +\varepsilon . \end{array}$$

Denote the following:

(23) $$\begin{array}{c} {\tilde{W}}_{r}=W_{r}-{\hat{W}}_{r}. \end{array}$$

Equation (22) can be written as follows:

(24) $$\begin{array}{c} f_{r}-{\hat{f}}_{r}={\tilde{W}}_{r}\phi +\varepsilon . \end{array}$$

An adaptive control law is constructed as follows:

(25) $$\begin{array}{c} \hat{W}=s\phi . \end{array}$$

### 3.3. Stability Analysis

**Theorem 1 .**

Based on the Lyapunov stability theory, for the ship dynamic model (4) with control input (19) and an adaptive control law (25), the tracking error of the system converges to zero and the system is asymptotically stable.

**Proof**

The Lyapunov function is constructed as follows:

(26) $$\begin{array}{c} V=\frac{1}{2}z_{1}^{2}+\frac{1}{2}s^{2}+\frac{1}{2}{\tilde{W}}_{r}^{T}{\tilde{W}}_{r}. \end{array}$$

Taking the derivative of (16) can obtain the following:

(27) $$\begin{array}{c} {\dot{z}}_{1}=\dot{\psi }-{\dot{\psi }}_{d}+\dot{\beta }. \end{array}$$

Substituting (17) into (27) can obtain the following:

(28) $$\begin{array}{c} {\dot{z}}_{1}=z_{2}-k_{1}z_{1}. \end{array}$$

The derivative of (23) can obtain the following:

(29) $$\begin{array}{c} {\overset{\cdot }{\tilde{W}}}_{r}={\dot{W}}_{r}-{\overset{\cdot }{\hat{W}}}_{r}=-\overset{\cdot }{\hat{W}}. \end{array}$$

Substituting (25) into (29) can obtain the following:

(30) $$\begin{array}{c} {\overset{\cdot }{\tilde{W}}}_{r}=-s\phi . \end{array}$$

Taking the derivative of (18) can obtain the following:

(31) $$\begin{array}{c} \dot{s}\left(t\right)=k_{2}{\dot{z}}_{1}+{\dot{z}}_{2}. \end{array}$$

Substituting fd28(28) into fd31(31) can obtain the following:

(32) $$\begin{array}{c} \dot{s}\left(t\right)=k_{2}\left(z_{2}-k_{1}z_{1}\right)+{\dot{z}}_{2}. \end{array}$$

Taking the derivative of (17) can obtain the following:

(33) $$\begin{array}{c} {\dot{z}}_{2}=\dot{r}+k_{1}{\dot{z}}_{1}-{\ddot{\psi }}_{d}+\ddot{\beta }. \end{array}$$

Substituting fd6(6) into fd33(33) can obtain the following:

(34) $$\begin{array}{c} {\dot{z}}_{2}=\frac{m_{11}-m_{22}}{m_{33}}uv-\frac{d_{33}}{m_{33}}r+\frac{1}{m_{33}}\tau_{r}+f_{r}+k_{1}{\dot{z}}_{1}-{\ddot{\psi }}_{d}+\ddot{\beta }. \end{array}$$

Substituting fd34(34) into fd32(32) can obtain the following:

(35) $$\begin{array}{c} \dot{s}\left(t\right)=k_{2}\left(z_{2}-k_{1}z_{1}\right)+\frac{m_{11}-m_{22}}{m_{33}}uv-\frac{d_{33}}{m_{33}}r+\frac{1}{m_{33}}\tau_{r}+f_{r}+k_{1}{\dot{z}}_{1}-{\ddot{\psi }}_{d}+\ddot{\beta }. \end{array}$$

Substituting fd19(19) into fd35(35) can obtain the following:

(36) $$\begin{array}{c} \dot{s}\left(t\right)=k_{2}\left(z_{2}-k_{1}z_{1}\right)+\frac{m_{11}-m_{22}}{m_{33}}uv-\frac{d_{33}}{m_{33}}r+\frac{d_{33}}{m_{33}}r-\frac{m_{11}-m_{22}}{m_{33}}uv-{\hat{f}}_{r}-k_{1}{\dot{z}}_{1}+{\ddot{\psi }}_{d}-\ddot{\beta }-z_{1}+k_{2}\left(k_{1}z_{1}-z_{2}\right)-k_{3}s-\eta \mathrm{sgn}\left(s\right)+f_{r}+k_{1}{\dot{z}}_{1}-{\ddot{\psi }}_{d}+\ddot{\beta }\\ =-{\hat{f}}_{r}-z_{1}-k_{3}s-\eta \mathrm{sgn}\left(s\right)+f_{r}. \end{array}$$

Substituting (24) into (36) can obtain the following:

(37) $$\begin{array}{c} \dot{s}\left(t\right)=-z_{1}-k_{3}s-\eta \mathrm{sgn}\left(s\right)+{\tilde{W}}_{r}\phi +\varepsilon . \end{array}$$

The derivative of (26) can obtain the following:

(38) $$\begin{array}{c} \dot{V}=z_{1}{\dot{z}}_{1}+s\dot{s}+{\tilde{W}}_{r}^{T}{\overset{\cdot }{\tilde{W}}}_{r}. \end{array}$$

Substituting (28), (30) and (37) into (38) can obtain the following:

(39) $$\begin{array}{c} \dot{V}=z_{1}\left(z_{2}-k_{1}z_{1}\right)+s\left(-z_{1}-k_{3}s-\eta \mathrm{sgn}\left(s\right)+{\tilde{W}}_{r}\phi +\varepsilon \right)-{\tilde{W}}_{r}^{T}s\phi \\ =-k_{1}z_{1}^{2}+z_{1}z_{2}-z_{1}s+\varepsilon s-k_{3}s^{2}-\eta \left|s\right|. \end{array}$$

Substituting (18) into (39) can obtain the following:

(40) $$\begin{array}{c} \dot{V}=-k_{1}z_{1}^{2}+z_{1}z_{2}-z_{1}\left(k_{2}z_{1}+z_{2}\right)-k_{3}s^{2}-\eta \left|s\right|+\varepsilon s\\ =-\left(k_{1}+k_{2}\right)k_{1}z_{1}^{2}-k_{3}s^{2}-\eta \left|s\right|+\varepsilon s. \end{array}$$

When the conditions are established:

(41) $$\begin{array}{c} \left|\varepsilon \right|\leq \eta . \end{array}$$

We have the following:

(42) $$\begin{array}{c} \dot{V}\leq -\left(k_{1}+k_{2}\right)k_{1}z_{1}^{2}-k_{3}s^{2}\\ \leq 0. \end{array}$$

Based on the Lyapunov stability theory, the tracking error converges to zero and the system is asymptotically stable.

## 4. Simulation Studies

### 4.1. Example Introduction and Parameter Configuration

In order to verify the control effect of the adaptive backstepping sliding mode control algorithm on the ship, a supply ship [30] in the literature is used. The length of the ship is 76.2 meters and the weight is 6000 tons. The relevant parameters of the ship are as follows: *m*_11_ = 1.1274, *m*_22_ = 1.8902, *m*_23_ = −0.0744, *m*_32_ = −0.0744, *m*_33_ = 0.1278, *d*_11_ = 0.0358, *d*_22_ = 0.1183, *d*_23_ = −0.0124, *d*_32_ = −0.0041, *d*_33_ = 0.0308. The initial position is [−2 m, 2 m, −*π*/2 rad]. The initial speed is [0 m/s, 0 m/s, 0.07 rad/s]. The desired position is [0 m, 0 m, 0 rad]. The desired speed is [0 m/s, 0 m/s, 0 rad/s].

The experiment was conducted in Intel(R) Core(TM) i7-3612QE CPU @ 2.10 GHz 2.10 GHz, 16.0 GB memory, and 64 bit OS.

The RBF neural network is composed of three layers, that is the input layer, the hidden layer, and the output layer. There are six nodes in the input layer, that is $\left[z_{1},{\dot{z}}_{1},z_{2},{\dot{z}}_{2},r,\dot{r}\right]$. There are seven nodes in the hidden layer, and the Gauss function is used as the radial basis function. There are one node in the output layer, that is ${\hat{f}}_{r}$, the approximation of *f*_*r*_ by RBF neural network. The initial weight of the RBF neural network is zero. The parameters of the radial basis function are set as follows:

(43) $$\begin{array}{c} \sigma_{j}=0.2,1\leq j\leq 7,\\ \mu =\left[\begin{array}{ccccccc} -1.5 & -1 & -0.5 & 0 & 0.5 & 1 & 1.5\\ -1.5 & -1 & -0.5 & 0 & 0.5 & 1 & 1.5\\ -1.5 & -1 & -0.5 & 0 & 0.5 & 1 & 1.5\\ -1.5 & -1 & -0.5 & 0 & 0.5 & 1 & 1.5\\ -1.5 & -1 & -0.5 & 0 & 0.5 & 1 & 1.5\\ -1.5 & -1 & -0.5 & 0 & 0.5 & 1 & 1.5 \end{array}\right]. \end{array}$$

### 4.2. Results without Disturbance

The initial forward position is −2 m, the initial sway position is 2 m, the initial yaw angle is −1.5 rad, the initial forward velocity is 0 m/s, the initial yaw velocity is 0 m/s, and the initial yaw rate is −1.5 rad/s. The desired forward position is set to 0 M. The desired sway position is 0 M. The expected yaw angle is 0 rad. The expected forward speed is 0 m/s. The desired yaw velocity is set to 0 m/s. The expected yaw rate is 0 rad/s.

**Figure 4:**
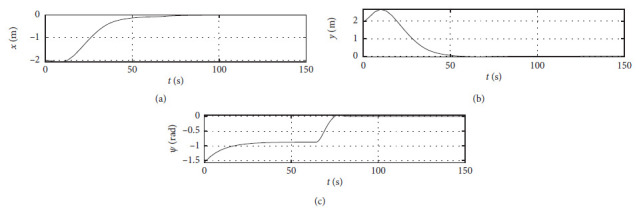
Ship position response curve.

Figure 4 shows the position response curve of the ship. The horizontal axis represents the time in seconds. The vertical axis in the upper subfigure shows the surge position with unit of m. The vertical axis in the middle subfigure shows the sway position with unit of m. The vertical axis in the lower subfigure shows the yaw angle with unit of rad.

**Figure 5:**
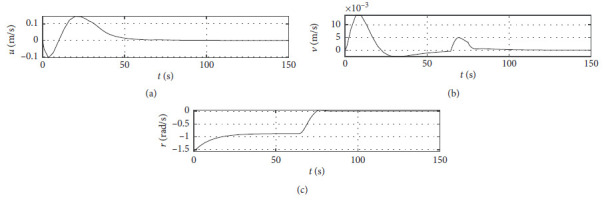
Speed velocity curve.

Figure 5 shows the ship speed response curves. The horizontal axis represents the time in seconds. The vertical axis in the middle subfigure shows the sway speed with unit of in m/s. The vertical axis in the lower subfigure shows the yaw angle speed with unit of in rad/s.

**Figure 6:**
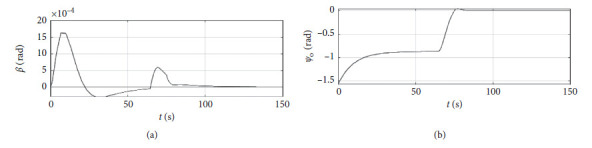
Shift angle and heading angle curve.

Figure 6 shows the shift angle and heading error curve of ship control. The horizontal axis represents the time in seconds. The vertical axis in the upper subfigure represents the shift angle in rad. The horizontal axis in the lower subfigure represents the heading error in rad.

**Figure 7:**
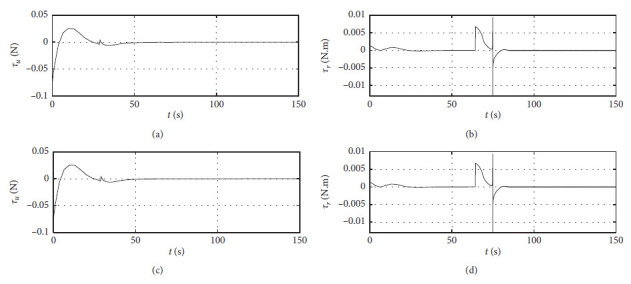
Control input curve.

Figure 7 shows the input curve of ship control. The horizontal axis represents the time in seconds. The vertical axis in the upper subfigure represents *τ*_*u*_ in N. The horizontal axis in the lower subfigure represents *τ*_*r*_ in N·m.

Figures 4–7 shows that the adaptive backstepping sliding mode control with a neural estimator can make the ship reach the desired position and attitude.

### 4.3. Results at Different Levels of Sea Conditions

#### 4.3.1. At the Third Level of Sea Conditions

**Figure 8:**
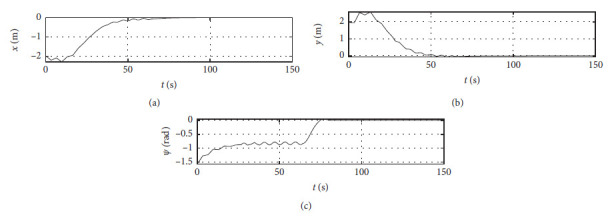
Position response at the third level of sea conditions.

The control algorithm is performed at the third level of sea conditions, and other conditions are the same as those in Section 4.2.  Figure 8 shows the position response at the third level of sea conditions. The horizontal axis represents the time in seconds. The vertical axis in the upper subfigure shows the surge position with unit of m. The vertical axis in the middle subfigure shows the sway position with unit of m. The vertical axis in the lower subfigure shows the yaw angle with unit of rad.

**Figure 9:**
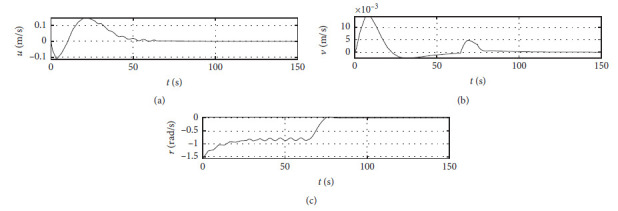
Speed response at the third level of sea conditions.

Figure 9 shows the ship speed response curve at the third level of sea condition. The horizontal axis represents the time in seconds. The vertical axis in the middle subfigure shows the sway speed with unit of in m/s. The vertical axis in the lower subfigure shows the yaw angle speed with unit of in rad/s.

**Figure 10:**
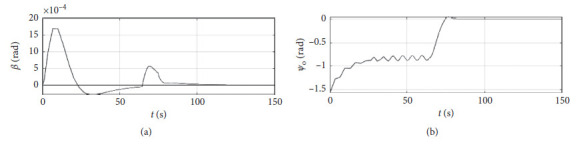
Shift angle and heading angle curve at the third level of sea conditions.

Figure 10 shows the shift angle and heading error curve of ship control. The horizontal axis represents the time in seconds. The vertical axis in the upper subfigure represents the shift angle in rad. The horizontal axis in the lower subfigure represents the heading error in rad.

**Figure 11:**
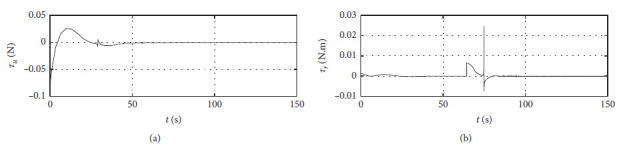
Control input at the third level of sea conditions.

Figure 11 shows the input curve of ship control. The horizontal axis represents the time in seconds. The vertical axis in the upper subfigure represents *τ*_*u*_ in N. The horizontal axis in the lower subfigure represents *τ*_*r*_ in N·m.

#### 4.3.2. At the Fifth Level of Sea Conditions

**Figure 12:**
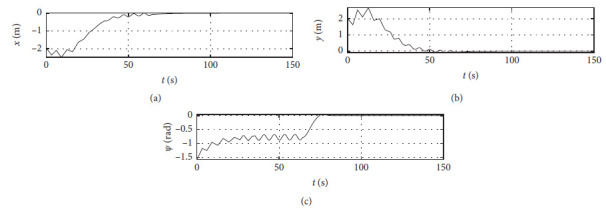
Position response at the fifth level of sea conditions.

The control algorithm is performed at the fifth level of sea conditions, and other conditions are the same as those in Section 4.2.  Figure 12 shows the position response at the fifth level of sea conditions. The horizontal axis represents the time in seconds. The vertical axis in the upper subfigure shows the surge position with unit of m. The vertical axis in the middle subfigure shows the sway position with unit of m. The vertical axis in the lower subfigure shows the yaw angle with unit of rad.

**Figure 13:**
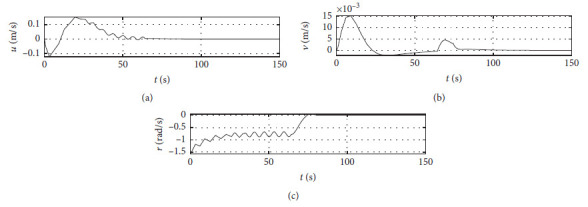
Speed response at the fifth level of sea condition.

Figure 13 shows the ship speed response curve at the fifth level of sea condition. The horizontal axis represents the time in seconds. The vertical axis in the middle subfigure shows the sway speed with unit of in m/s. The vertical axis in the lower subfigure shows the yaw angle speed with unit of in rad/s.

**Figure 14:**
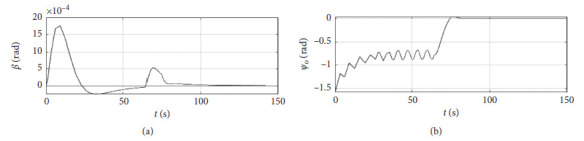
Shift angle and heading angle curve at the fifth level of sea conditions.

Figure 14 shows the shift angle and heading error curve of ship control. The horizontal axis represents the time in seconds. The vertical axis in the upper subfigure represents shift angle in rad. The horizontal axis in the lower subfigure represents the heading error in rad.

**Figure 15:**
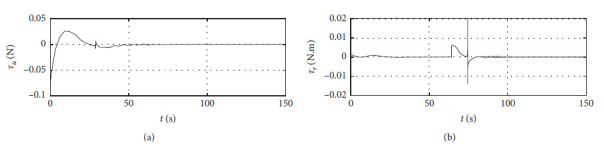
Control input at the fifth level of sea conditions.

Figure 15 shows the input curve of ship control. The horizontal axis represents the time in seconds. The vertical axis in the upper subfigure represents *τ*_*u*_ in N. The horizontal axis in the lower subfigure represents *τ*_*r*_ in N·m.

Figures 8–15 show that the designed RBF neural network observer can track both the mild level 3 sea state and the bad level 5 sea state, although the wave disturbance has relatively fast time-varying disturbance. The algorithm has good tracking performance and can realize the accurate estimation of wave disturbance, especially in bad sea conditions.

### 4.4. Results considering Ship-Bank Interaction Effect

**Figure 16:**
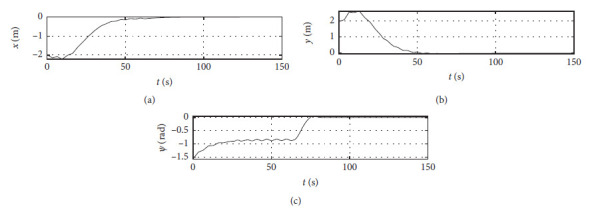
Position response with ship-bank interaction.

The Ship-bank interaction effect is considered, and other conditions are the same as those in Section 4.2.  Figure 16 shows the position response with the ship-bank interaction effect. The horizontal axis represents the time in seconds. The vertical axis in the upper subfigure shows the surge position with unit of m. The vertical axis in the middle subfigure shows the sway position with unit of m. The vertical axis in the lower subfigure shows the yaw angle with unit of rad.

**Figure 17:**
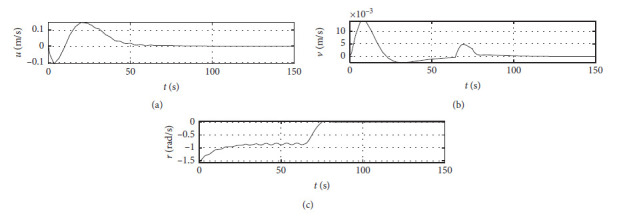
Speed response with ship-bank interaction.

Figure 17 shows the ship speed response curve with ship-bank interaction. The horizontal axis represents the time in seconds. The vertical axis in the middle subfigure shows the sway speed with unit of in m/s. The vertical axis in the lower subfigure shows the yaw angle speed with unit of in rad/s.

**Figure 18:**
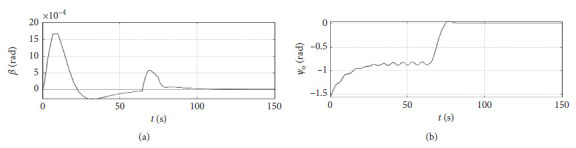
Shift angle and heading angle with ship-bank interaction.

Figure 18 shows the shift angle and heading error curve of ship control. The horizontal axis represents the time in seconds. The vertical axis in the upper subfigure represents the shift angle in rad. The horizontal axis in the lower subfigure represents the heading error in rad.

**Figure 19:**
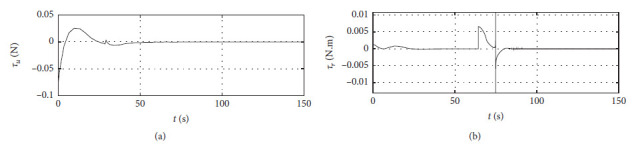
Control input with ship-bank interaction.

Figure 19 shows the input curve of ship control. The horizontal axis represents the time in seconds. The vertical axis in the upper subfigure represents *τ*_*u*_ in N. The horizontal axis in the lower subfigure represents *τ*_*r*_ in N·m.

Figures 16–19 show that the adaptive backstepping sliding mode control with a neural estimator can make the ship reach the desired position and attitude under disturbance.

### 4.5. Results with Different Parameters of Ship

Different parameters of a ship are also tested, and other conditions are the same as those in Section 4.4. A mariner class vessel [30] is used. The length of the ship is 160.93 meters. The relevant parameters of the ship are as follows: *m*_11_ = 0.0084, *m*_22_ = 0.0155, *m*_23_ = −0.00009, *m*_32_ = −0.00023, *m*_33_ = 0.00083, *d*_11_ = 0.0018, *d*_22_ = 0.0116, *d*_23_ = 0.0050, *d*_32_ = 0.0026, *d*_33_ = 0.0017.

**Figure 20:**
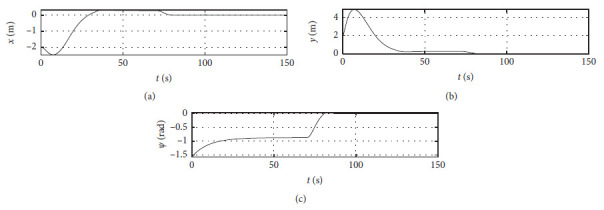
Position response of a mariner class vessel.

Figure 20 shows the position response of a mariner class vessel. The horizontal axis represents the time in seconds. The vertical axis in the upper subfigure shows the surge position with unit of m. The vertical axis in the middle subfigure shows the sway position with unit of m. The vertical axis in the lower subfigure shows the yaw angle with unit of rad.

**Figure 21:**
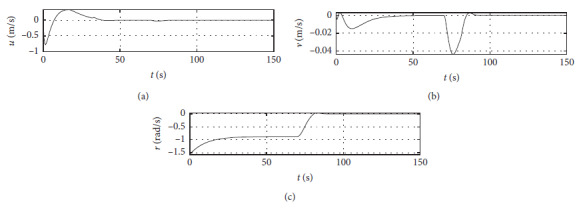
Speed response of a mariner class vessel.

Figure 21 shows the ship speed response curve of a mariner class vessel. The horizontal axis represents the time in seconds. The vertical axis in the middle subfigure shows the sway speed with unit of in m/s. The vertical axis in the lower subfigure shows the yaw angle speed with unit of in rad/s.

**Figure 22:**
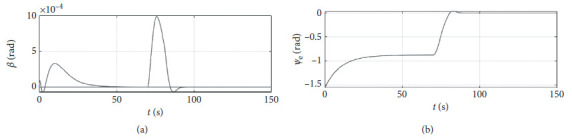
Shift angle and heading angle of a mariner class vessel.

Figure 22 shows the shift angle and heading error curve of ship control of a mariner class vessel. The horizontal axis represents the time in seconds. The vertical axis in the upper subfigure represents the shift angle in rad. The horizontal axis in the lower subfigure represents the heading error in rad.

**Figure 23:**
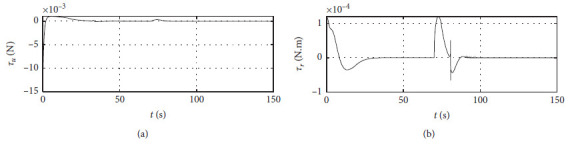
Control input of a mariner class vessel.

Figure 23 shows the input curve of ship control. The horizontal axis represents the time in seconds. The vertical axis in the upper subfigure represents *τ*_*u*_ in N. The horizontal axis in the lower subfigure represents *τ*_*r*_ in N·m.

Figures 20–23 show that the adaptive backstepping sliding mode control with neural estimator can deal with ships of different parameters considering drift angle and ship-bank interaction.

### 4.6. Results with Different Initial Conditions

**Figure 24:**
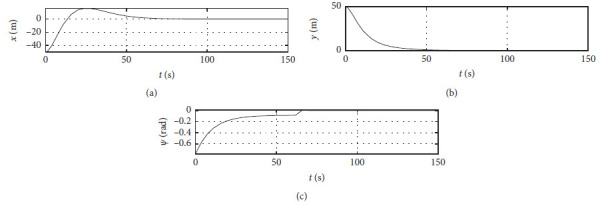
Position response with different initial conditions.

Different initial conditions are also tested, and other conditions are the same as those in Section 4.4. The initial position is [−50 m, 50 m, −*π*/4 rad]. The initial speed is [0 m/s, 0 m/s, 0.07 rad/s]. The desired position is [0 m, 0 m, 0 rad]. The desired speed is [0 m/s, 0 m/s, 0 rad/s].  Figure 24 shows the position response with different initial conditions. The horizontal axis represents the time in seconds. The vertical axis in the upper subfigure shows the surge position with unit of m. The vertical axis in the middle subfigure shows the sway position with unit of m. The vertical axis in the lower subfigure shows the yaw angle with unit of rad.

**Figure 25:**
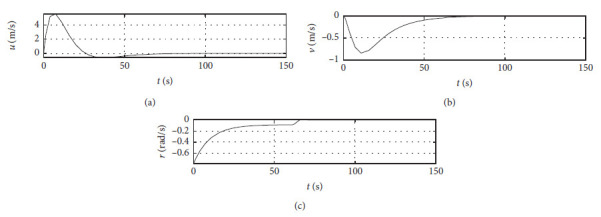
Speed response with different initial conditions.

Figure 25 shows the ship speed response curve with different initial conditions. The horizontal axis represents the time in seconds. The vertical axis in the middle subfigure shows the sway speed with unit of in m/s. The vertical axis in the lower subfigure shows the yaw angle speed with unit of in rad/s.

**Figure 26:**
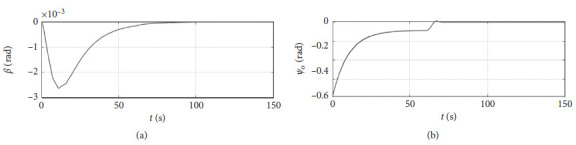
Shift angle and heading angle with different initial conditions.

Figure 26 shows the shift angle and heading error curve of ship control. The horizontal axis represents the time in seconds. The vertical axis in the upper subfigure represents shift angle in rad. The horizontal axis in the lower subfigure represents the heading error in rad.

**Figure 27:**
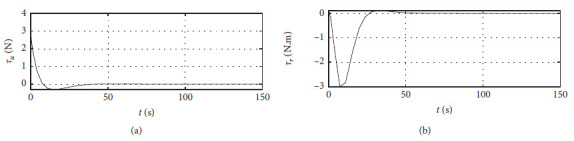
Control input with different initial conditions.

Figure 27 shows the input curve of ship control. The horizontal axis represents the time in seconds. The vertical axis in the upper subfigure represents *τ*_*u*_ in N. The horizontal axis in the lower subfigure represents *τ*_*r*_ in N·m.

Figures 24–27 show that the adaptive backstepping sliding mode control with neural estimator can deal with ships with different initial conditions considering drift angle and ship-bank interaction.

### 4.7. Performance Comparison

**Figure 28:**
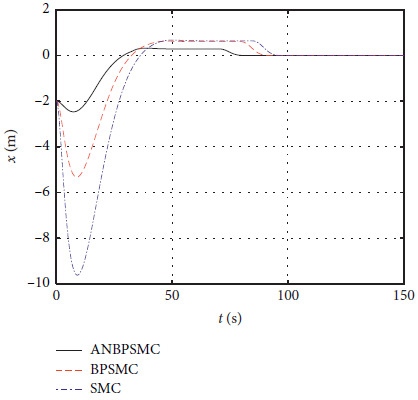
Surge response of different algorithms.

In order to verify the effectiveness of the algorithm, the control effects of different algorithms are compared, including basic sliding mode control (SMC), backstepping sliding mode control (BPSMC), and adaptive neural backstepping sliding mode control (ANBPSMC). The remaining parameters remain unchanged as in Section 4.4.  Figure 28 shows the surge response of different algorithms.

**Figure 29:**
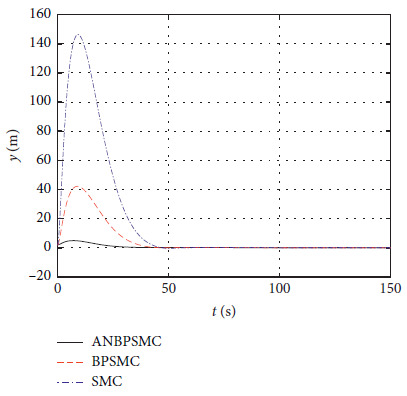
Sway response of different algorithms.

Figure 29 shows the sway response of different algorithms.

**Figure 30:**
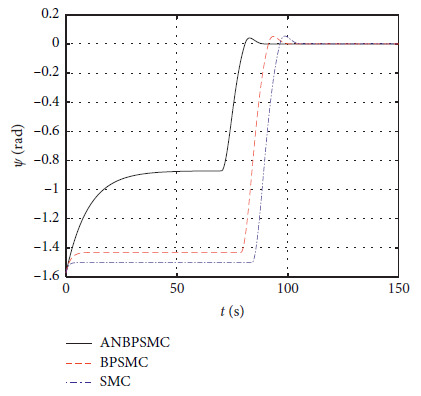
Yaw response of different algorithms.

Figure 30 shows the yaw response of different algorithms.

**Figure 31:**
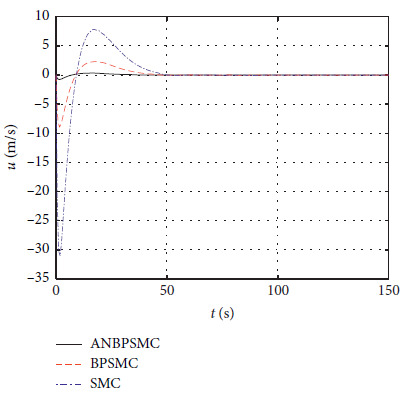
Surge speed response of different algorithms.

Figure 31 shows the surge speed response of different algorithms.

**Figure 32:**
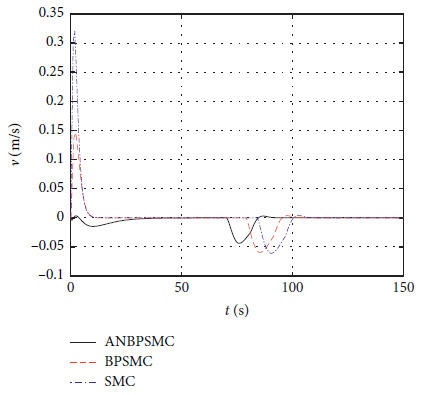
Sway speed response of different algorithms.

Figure 32 shows the sway speed response of different algorithms.

**Figure 33:**
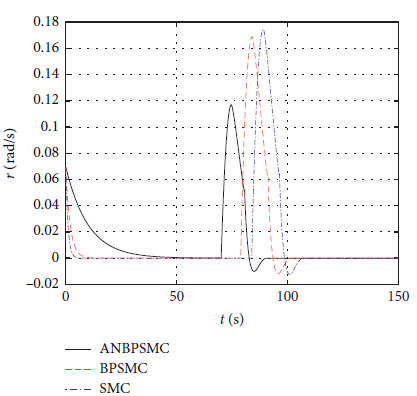
Yaw angle speed response of different algorithms.

Figure 33 shows the yaw angle speed response of different algorithms.

**Figure 34:**
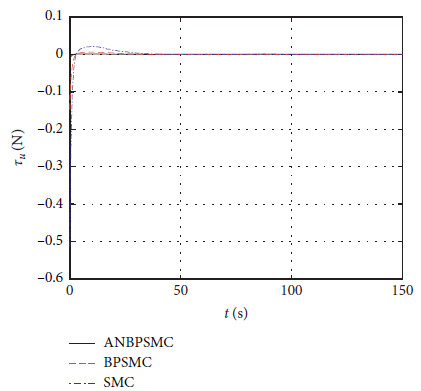
Input *τ*_*u*_ curve of different algorithms.

Figure 34 shows the input *τ*_*u*_ curve of different algorithms.

**Figure 35:**
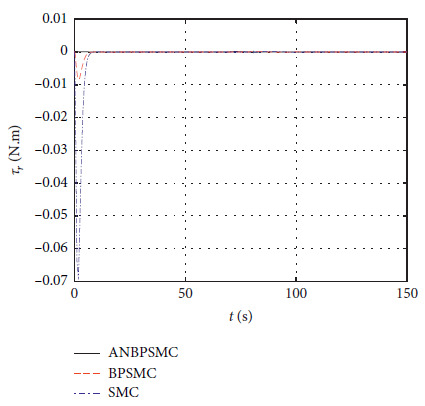
Input *τ*_*r*_ curve of different algorithms.

Figure 35 shows the input *τ*_*r*_ curve of different algorithms.

Figures 28–35 show that, compared with the basic sliding mode control algorithm, the adaptive backstepping sliding mode control with neural estimator has smaller overshoot, shorter system regulation time, smaller heading angle error, and smaller control torque.

## 5. Conclusion

This paper presents an adaptive backstepping sliding mode control algorithm with a neural estimator for underactuated ships considering both ship-bank interaction effect and shift angle. Based on the feature that the RBF neural network can approach any function, the unknown parameters of the ship model and environmental disturbance are estimated. Based on the Lyapunov stability theory, the tracking error will converge to zero and the system is asymptotically stable. The design of the switching function makes the system robust to uncertainties and external disturbances and avoids chattering. The results show that compared with the basic sliding mode control algorithm and backstepping sliding mode control, the neural estimator adaptive backstepping sliding mode control has less overshoot, shorter system regulation time, less heading error, and less control torque of the actuator. The technical contributions of the control method are summarized as follows:

1. The Ship-bank interaction effect is considered in underactuated ship motion control, especially in berthing, crossing bridge, and near-shore navigation.
2. The time-varying shift angle is estimated by a radial basis function (RBF) neural network with weight parameter adaptive law in an underactuated ship heading control.
3. An adaptive backstepping sliding mode control with a neural estimator is designed. Based on the Lyapunov stability theory, the tracking error converges to zero and the system is asymptotically stable.

The next step is to improve the control algorithm to improve the control accuracy and robustness of the underactuated ship motion control.

## Data Availability

The data used to support the findings of this study are included within the article.

## References

[1] Yu Z., Bao X., Nonami K. (2008). Course keeping control of an autonomous boat using low cost sensors. *Journal of System Design and Dynamics*.

[2] Liu Z., Chu R. (2020). Robust adaptive heading control for a surface vessel with drift angles. *Ocean Engineering*.

[3] Lu X. Y., Liu Z. Q., Chu Z. Z. (2020). Robust adaptive heading control for underactuated ships with sideslip angle amendment. *Journal of Shanghai Maritime University*.

[4] Zhuo S. (2019). *Path following Control of Underactuated Unmanned Vehicles with Time-Varying*.

[5] Lataire E., Vantorre M. Ship-bank interaction induced by irregular bank geometries.

[6] Chu Y., Fei J., Hou S. (2019). Adaptive neural backstepping PID global sliding mode fuzzy control of MEMS gyroscope. *IEEE Access*.

[7] Wang R., Miao K., Sun J., Li J., Chen D. (2019). Intelligent control algorithm for USV with input saturation based on RBF network compensation. *International Journal of Reasoning-Based Intelligent Systems*.

[8] Liu C., Zou Z. J., Wang X. G. Path following and stabilization of underactuated surface ships based on hierarchical sliding mode.

[9] Li F., Hu J., Wang J., Wang T. (2017). Adaptive backstepping sliding mode control for a class of uncertain nonlinear systems with input constraints. *Xi Tong Gong Cheng Yu Dian Zi Ji Shu/Systems Engineering and Electronics*.

[10] Chen N., Song F., Li G., Sun X., Ai C. (2013). An adaptive sliding mode backstepping control for the mobile manipulator with nonholonomic constraints. *Communications in Nonlinear Science and Numerical Simulation*.

[11] Wang R. (2018). *Adaptive Sliding Mode Control for Trajectory Tracking of Underactuated Ship*.

[12] Meng W. J., Guo C., Yu Y. Sliding-mode robust tracking control for an underactuated ship with nonzero off-diagonal terms.

[13] Wang R., Miao K., Zhao Y., Deng H., Sun J., Du J. (2020). Robust sliding mode control of ship based on neural network under uncertain conditions. *Advances in Intelligent Systems and Computing*.

[14] Wen Y., Ren X. (2012). Neural observer-based adaptive compensation control for nonlinear time-varying delays systems with input constraints. *Expert Systems with Applications*.

[15] Sun H., Hou L., Zong G., Yu X. (2020). Adaptive decentralized neural network tracking control for uncertain interconnected nonlinear systems with input quantization and time delay. *IEEE Transactions on Neural Networks and Learning Systems*.

[16] Cheng C., Zhang Y., Liu S. (2019). Neural observer-based adaptive prescribed performance control for uncertain nonlinear systems with input saturation. *Neurocomputing*.

[17] Gajate A., Haber R. E., Vega P. I., Alique J. R. (2010). A transductive neuro-fuzzy controller: application to a drilling process. *IEEE Transactions on Neural Networks*.

[18] Martin A. G., Guerra R. E. H. (2009). Internal model control based on a neurofuzzy system for network applications. a case study on the high-performance drilling process. *IEEE Transactions on Automation Science and Engineering*.

[19] Kelly R., Haber R., Haber-Guerra R. E., Reyes F. (1999). Lyapunov stable control of robot manipulators: a fuzzy self-tuning procedure. *Intelligent Automation & Soft Computing*.

[20] Ramírez M., Haber R., Peña V., Rodríguez I. (2004). Fuzzy control of a multiple hearth furnace. *Computers in Industry*.

[21] Guerra R. H., Quiza R., Villalonga A., Arenas J., Castano F. (2019). Digital twin-based optimization for ultraprecision motion systems with backlash and friction. *IEEE Access*.

[22] Guo X., Liang Z., Li C. (2018). Finite time tracking control of mobile robot based on non-singular fast terminal sliding mode. *Systems Science & Control Engineering*.

[23] Elmokadem T., Zribi M., Youcef-Toumi K. (2018). Control for dynamic positioning and way-point tracking of underactuated autonomous underwater vehicles using sliding mode control. *Journal of Intelligent & Robotic Systems*.

[24] Sun Z., Zhang G., Yi B., Zhang W. (2017). Practical proportional integral sliding mode control for underactuated surface ships in the fields of marine practice. *Ocean Engineering*.

[25] Wang Y. L. (2015). *Research of Nonlinear Sliding Mode Control for Underactuated Surface Vessel Trajectory Tracking*.

[26] Dai C. S. (2017). *Research on Adaptive Iterative Sliding Mode Control for Underactuated Ship Motion*.

[27] Jiang-hua S., Zhang He Neural network sliding mode path following control based on the DVS algorithm of underactuated ships.

[28] Zou T. Y., Shen Z. P., Dai C. S. Adaptive iterative sliding mode berthing control for underactuated ship based on chaotic particle swarm.

[29] Gao S. J. (2019). Ship-bank interaction effect and ship maneuvering security. *Tianjin Navigation*.

[30] Fossen T. I., Sagatun S. I., Sørensen A. J. (1996). Identification of dynamically positioned ships. *Control Engineering Practice*.

